# Direct Printing of Stretchable Elastomers for Highly Sensitive Capillary Pressure Sensors

**DOI:** 10.3390/s18041001

**Published:** 2018-03-28

**Authors:** Wenguang Liu, Chaoyi Yan

**Affiliations:** 1 School of Advanced Materials, Shenzhen Graduate School, Peking University, Shenzhen 518055, China; 1501213833@pku.edu.cn; 2 College of Chemistry and Environmental Engineering, Shenzhen University, Shenzhen 518060, China

**Keywords:** 3D printing, stretchable electronics, pressure sensor, PDMS, conductive elastomer

## Abstract

We demonstrate the successful fabrication of highly sensitive capillary pressure sensors using an innovative 3D printing method. Unlike conventional capacitive pressure sensors where the capacitance changes were due to the pressure-induced interspace variations between the parallel plate electrodes, in our capillary sensors the capacitance was determined by the extrusion and extraction of liquid medium and consequent changes of dielectric constants. Significant pressure sensitivity advances up to 547.9 KPa^−1^ were achieved. Moreover, we suggest that our innovative capillary pressure sensors can adopt a wide range of liquid mediums, such as ethanol, deionized water, and their mixtures. The devices also showed stable performances upon repeated pressing cycles. The direct and versatile printing method combined with the significant performance advances are expected to find important applications in future stretchable and wearable electronics.

## 1. Introduction

Stretchable electronics represent a new type of soft electronics that can deform into arbitrary shapes and conform to complex non-planar surfaces to achieve unprecedented applications [1,2]. Over the years, many efforts have been devoted to the developments of innovative stretchable electronic devices, ranging from basic stretchable structure and material design [3,4,5,6,7,8] to the demonstration of individual stretchable electronic units [5,6] and to the fabrication of integrated complex stretchable electronic systems [9,10,11] for practical applications. Among them, pressure sensors have received great attention as important interactive electronic devices for future applications, such as human motion detection [12,13] and human-machine interaction [14,15].

Several types of device structures, such as capacitive [6,7,13,14,16,17,18,19,20,21,22], resistive [12,23,24], transistor [20,25,26,27] and optical types [28] have been successfully demonstrated for pressure sensing applications, with capacitive type as the dominant device structure due to its simple device fabrication process, fast response rate and excellent device stability. Most capacitive pressure sensors in previous works employed analogous sensing mechanisms where the applied pressure affects the interspace between the parallel plate electrodes and hence device capacitance. The device performance, especially pressure sensitivity, could be effectively improved through the design of innovative structures for the dielectric layer. For example, Bao group [20] demonstrated the fabrication of inversely templated polydimethylsiloxane (PDMS) micro-pyramids as dielectric layer between two stretchable carbon nanotube (CNT) electrodes. The pressure sensitivity was successfully improved from 0.02 KPa^−1^ for unstructured PDMS film to 0.55 KPa^−1^ for PDMS micro-pyramids. Similar structure designs were extensively employed to improve the pressure sensitivity of pressure sensors in following studies [17,24,29,30].

However, developments of facile and scalable fabrication techniques and further improvements of the sensing performances of pressure sensors are imperative for practical applications. In this report, we employed 3D printing technique to directly print the stretchable conductive inks for capillary pressure sensors. 3D printing can conveniently fabricate complex electrode patterns and greatly simplify the fabrication processes as compared to previous lithographic [8,31] and templated methods [6,18]. Moreover, the capillary pressure sensors in our work employ distinct pressure sensing mechanism where the capacitance variations were induced by the extrusion and extraction of liquid medium within the capillary channel. Unlike the capacitance variations based on interspace changes in previously reported devices, the changes of dielectric medium in our innovative designs leads to significant capacitance variations upon pressing. We achieved an exciting pressure sensitivity of 547.9 KPa^−1^. The direct and facile printing method we used is a versatile method, which can be readily extended for the fabrication of other stretchable electronic devices for practical applications.

## 2. Materials and Methods

### 2.1. Preparation of the CNT-PDMS Printable Ink

First, 3 g multi-wall carbon nanotubes (MWCNTs) (Time Nano, Chengdu, China, diameter 5–8 nm, length 10–30 μm, purity > 98%) was added into 50 g ethanol and dispersed using a mechanical homogenizer for 10 min (T10 Ultra-Turrax, IKA, Staufen im Breisgau, Germany). Then 32.7 g PDMS base (Sylgard 184, Dow Corning, Midland, MI, USA) was added into the suspension and mechanically homogenized for 30 min. After this, the solution was heated at 80 °C for 5 h to completely remove the ethanol content. Finally, 3.3 g of PDMS curer was added into the slurry and stirred for 10 min to get the printable ink.

### 2.2. 3D Printing Process

The capillary electrode patterns were designed using Solidworks 2016 (Waltham, MA, USA). The 3D printing processes were controlled using a commercial software (Cura, Ultimaker, Geldermalsen, The Netherlands) and carried out on a home-build 3D printer with mechanical extrusion rod. A printing nozzle with inner diameter of 0.4 mm was used. Height of the elastomer electrodes were set at 2 mm and the interspace between the two electrodes were 1 mm. The as-printed elastomer electrodes were cured at 80 °C for 1 h.

### 2.3. Pressure Sensor Assembly and Characterizations

Top and bottom of the elastomer electrodes were sealed using PDMS thin film (0.5 mm thick). Pre-molded PDMS container with injection tubing was used as solution pool and connected to the capillary electrodes. Morphology and microstructures of the elastomer electrodes were characterized using scanning electron microscopy (SEM, Zeiss Supra, Oberkochen, Germany). The capacitances were measured using a Keysight LCR meter (E4980AL, Agilent Technologie, Palo Alto, CA, USA).

## 3. Results and Discussion

**Figure 1:**
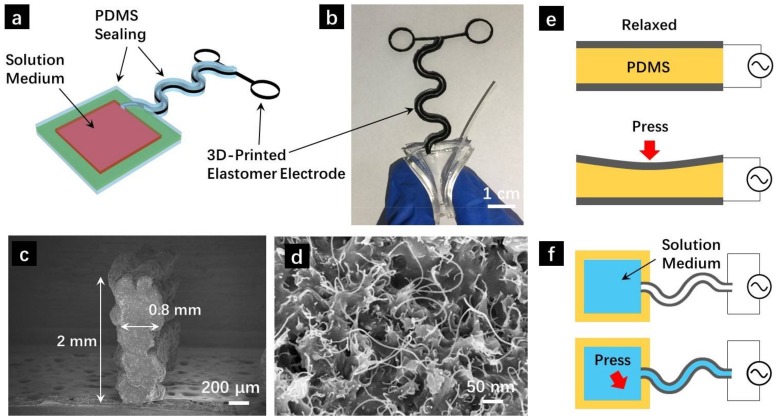
(**a**) Schematic diagram of the 3D printed capillary pressure sensors; (**b**) Photograph of the sensor showing soft PDMS solution pool; (**c**) Cross-sectional SEM image of the conductive elastomer electrode; (**d**) High-magnification SEM showing the uniform CNT distribution within the PDMS matrix; Structure and sensing mechanism of (**e**) conventional pressure sensor through the change of interspace between parallel electrodes and (**f**) our capillary pressure sensors through the change of dielectric medium.

Schematic diagram of the 3D printed capillary pressure sensor is shown in Figure 1a. The serpentine electrodes were designed in Solidworks 2016 and directed printed on temper glass substrate (Figures S1 and S2, Supplementary Materials) using a home-build 3D printer. All related parameters of the conductive elastomer electrodes can be conveniently tuned through software design and directed reflected in the as-printed patterns. The conductive ink was based on CNT/PDMS precursor composite with optimized formulation as demonstrated in our previous report [32,33]. The as-printed elastomer electrodes were cured at 80 °C for 1 h to get the solidified stretchable electrodes. Then the top and bottom surfaces of the electrodes were sealed using bare PDMS to get the capillary sensing channel. A pre-molded PDMS container was attached to the capillary electrodes and used as solution pool to store the liquid medium.

A typical SEM image showing the cross-section of the 3D printed elastomer electrode is shown in Figure 1c. With a nozzle diameter of 0.4 mm and 5-layer design, the printed electrode (as capillary wall) has an average width of 0.8 mm and height of 2 mm. An enlarged view of the electrode cross-section is shown in Figure 1d, where the CNT conductive filler can be found to distribute uniformly within the PDMS elastomer matrix, enabling good conductivity as well as stretchability of the elastomer electrodes.

Note that the sensing mechanism of our pressure sensors are distinct from previous research works. Most capacitive pressures sensors in previous works have standard parallel plate structure as shown in Figure 1e. Stretchable conductive electrodes, such as percolating CNT networks [3,4,5] and embedded Ag nanowire stretchable film [6,7,10,31] were used as the soft electrodes. The capacitance is determined via *C* = *εε*_0_*A*/*d*, where *ε* is the relative dielectric constant, *ε*_0_ is the dielectric permittivity of vacuum, *A* is the overlapping area and *d* is the vertical distance between the parallel plates. In those conventional designs, pressure was detected via the change of vertical distance *d* and hence overall capacitance *C* (Figure 1e). However, the pressure detection is achieved through the change of relative dielectric constant in our innovative structural design (Figure 1f). Upon applying pressure, the solution medium was extruded from the solution pool and partially filled the capillary tube. Significant capacitance changes can be detected due to the abrupt variation of relative dielectric constant *ε* (1 for air, 24.3 for ethanol at 25 °C).

**Figure 2:**
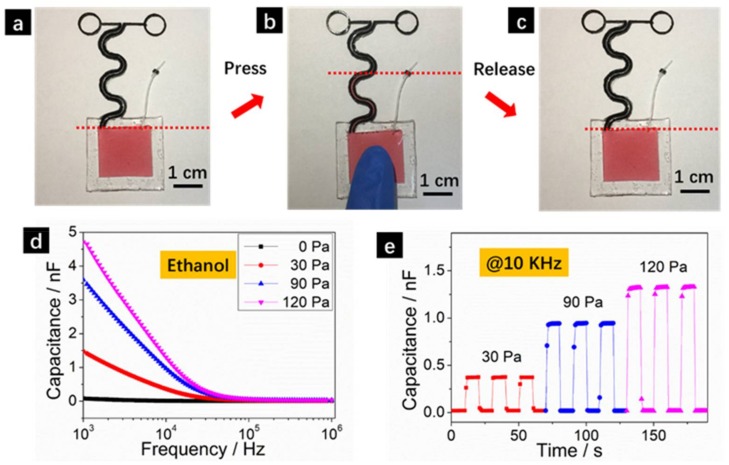
(**a**–**c**) Photographs of the pressure sensor upon pressing and releasing with dash lines indicating the liquid levels; Relationship between (**d**) capacitance-frequency and (**e**) capacitance-time at respective applied pressures; The solution medium used is ethanol (>99%) and the frequency was fixed at 10 KHz in (**e**).

The sensing behavior of our capillary pressure sensors is shown in Figure 2. Photographs showing the liquid level variations upon pressing and releasing are shown in Figure 2a–c. The liquid medium used is high purity ethanol (>99%) but with 1 wt. % Co(NO_3_)_2_·6H_2_O as dye to make the solution more visible in the photographs (no dye was used for consequent performance testing). Upon pressing, the liquid was extruded from the solution pool into the capillary sensing tube made of conductive elastomers (Figure 2b). The measured capacitance thus increased significantly due to the increase of dielectric constant as discussed earlier. Upon releasing, the extruded solution will automatically and fully retreat into the solution pool due to the intrinsic resilience of the PDMS elastomer. Enlarged views of the liquid levels can be found in Figure S3, Supplementary Materials.

The relationship between capacitance and frequency at applied pressures of 0 Pa, 30 Pa, 90 Pa and 120 Pa using ethanol as liquid medium are shown in Figure 2d. The frequency of the applied voltage was set within 1 KHz–1 MHz. The capacitance increased significantly at higher pressure since the extruded ethanol filled a larger portion of the capillary tube. For example, at the frequency of 1 KHz, the measured capacitance was 0.08 nF at relaxed state (0 Pa) and increased to 1.50 nF, 3.56 nF and 4.92 nF at the pressures of 30 Pa, 90 Pa and 120 Pa, respectively. There are also significant increases at the high frequency end as can be more clearly viewed in logarithm scale plot (Figure S4, Supplementary Materials). At a certain fixed pressure, the measured capacitance decreases at higher frequency. This can be attributed to the fact that the device capacitance is known to be related to applied alternative current (AC) signals via *C* ∝ 1/2π*f*, where *f* is the frequency. The dynamic sensing behaviors of the pressure sensors are shown in Figure 2e. The capacitance increased abruptly within 1 s when pressure was applied, indicating an excellent response rate. Moreover, the capacitance can fully restore to its initial value when the pressure was released and showed minimum delay due to the elastomer resilience properties as observed in previous reports [20]. The fast response rate is a characteristic property of capacitive pressure sensors as also observed in previous reports [21], making our capillary pressure sensors ideal candidates especially for the effective detections of dynamic pressure variations.

**Figure 3:**
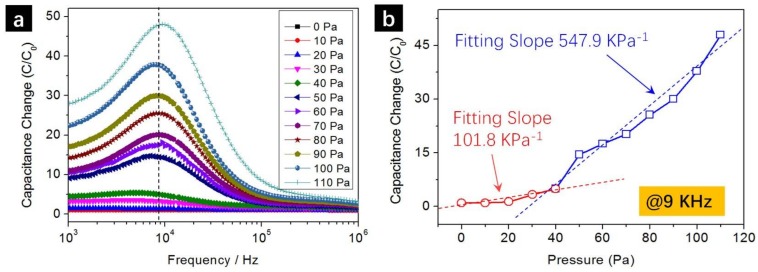
(**a**) Capacitance change *C*/*C*_0_ versus frequency curves at different applied pressures within 0–110 Pa; (**b**) Capacitance change *C*/*C*_0_ versus pressure curves and their linear fitting results showing the pressure sensitivity as high as 547.9 KPa^−1^.

Pressure sensitivity is one of the most important characteristics of pressure sensors and is studied in Figure 3. We performed a more detailed study using ethanol as liquid medium within the pressure range of 0–110 Pa (pressure increase step 10 Pa). The maximum pressure was set at 110 Pa since the capillary tube was fully filled with ethanol at this pressure. The pressure detection range can be effectively tuned by elongating the capillary tube length or design more compact capillary designs, such as bi-spiral patterns as shown in Figure S5, Supplementary Materials. Capacitance change is defined as *C*/*C*_0_, where *C* is the capacitance at respective applied pressures and *C*_0_ is the capacitance at 0 Pa. While a general increasing trend of capacitance at higher pressures was observed, the peak values of capacitance changes *C*/*C*_0_ were observed at the frequency of 9 KHz for all applied pressures (indicated by a dash line in Figure 3a). The plot of capacitance changes (values at 9 KHz) versus pressure is shown in Figure 3b. Relative slower capacitance increases were observed within 0–40 Pa (red circles in Figure 3b, linear fitting slope 101.8 KPa^−1^) but a larger slope was observed within the pressure range of 40–110 Pa (blue squares in Figure 3b, linear fitting slope 547.9 KPa^−1^).

It is worth mentioning that the pressure sensitivity of 547.9 KPa^−1^ represents a significant advance as compared to previous research works, as summarized in Table 1. For example, Dong et al. [34] reported the design of all-graphene pressure sensor integrated in electronic skin but the pressure sensitivity was only 0.002 KPa^−1^. Mannsfeld et al. [20] reported the fabrication of a standard conventional parallel-plate capacitive pressure sensor composed of two indium tin oxide on polyethylene terephthalate (ITO-PET) electrodes and an unstructured PDMS dielectric layer. The pressure sensitivity is as low as 0.02 KPa^−1^. Then the authors designed and fabricated microscale line-structured and pyramid-structured dielectric layers and successfully improved the sensitivity to 0.1 KPa^−1^ and 0.55 KPa^−1^, respectively. The micro-pyramid and analogous structures were extensively adopted in many following studies and the sensitivities were further improved through the optimization of micro-pyramid designs [24] and nanoelectrode innovations [11]. For example, Bae et al. [23] successfully improved the sensitivity to 14 KPa^−1^ through the fabrication of monolayer graphene coated PDMS microdot arrays for pressure sensors. However, most previous works employed the conventional device structure where the capacitance changes were due to the pressure-induced interspace *d* variations between the parallel plate electrodes (*C* = *εε*_0_*A*/*d*). Our innovative 3D printed capillary pressure sensor, on the contrary, employed a distinct concept by relating the pressure detection to solution medium extrusion/extraction in the capillary channel, leading to significant pressure sensitivity improvements. Enhanced pressure sensitivities were observed through the change of dielectric layer contact conditions with electrodes. Park et al. presented flexible capacitive pressure sensors that incorporate micropatterned pyramidal ionic gels to enable sensitivity of 41 KPa^−1^ [35]. It is also worth mentioning that Pan et al. fabricated an iontronic microdroplet array device consist of an array of nanoliter droplets sandwiched between two polymeric membranes, which achieved a sensitivity of 0.43 nF KPa^−1^ [36] (note that this is absolute capacitance change ΔC instead of relative capacitance change *C*/*C*_0_ adopted in Table 1); the authors also reported an electrospun ionic fabric utilizing nanofibrous structures achieving a sensitivity of 114 nF KPa^−1^ [37]. In above-mentioned devices using homogeneous material as the dielectric material, the capacitance only increases 2 times even when the dielectric layer was heavily squeezed to half its original thickness. However, we observed a 47.96 times capacitance increase only at a low pressure of 110 Pa (Figure 3) due to the large dielectric constant changes between ethanol and air as well as the frequency enhancement effect (peak value at 9 KHz). This pressure sensitivity can be further improved through device structure optimizations, such as using thinner capillary channels and more detailed studies are undergoing.

**Figure 4:**
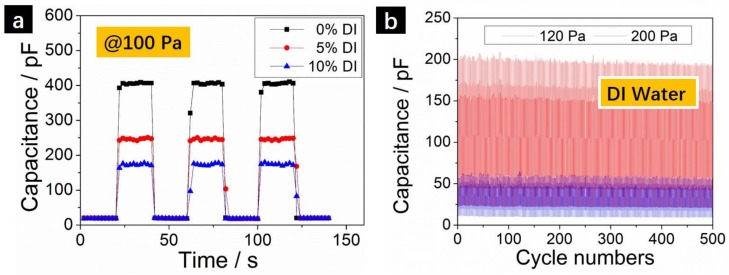
(**a**) The dynamic capacitance changes at 100 Pa using ethanol with 0%, 5% and 10% DI water as liquid medium; (**b**) Device stability testing using DI water as liquid medium at 120 Pa and 200 Pa pressures for 500 pressing cycles. The frequency was fixed at 10 KHz for (**a**,**b**).

Our innovative capillary pressure sensors can adopt different types of solution mediums and can all function properly as shown in Figure 4. Ethanol, DI water and their mixtures were all demonstrated to give analogous pressure sensing performances. Representative dynamic sensing behaviors using ethanol mixed with different volume percent of DI water are shown in Figure 4a. When applying a fixed pressure of 100 Pa, the capacitance increased to 0.41 nF when using pure ethanol as liquid medium. However, the absolute capacitances at pressed state were found to drop to 0.25 nF and 0.17 nF when ethanol was mixed with 5 vol. % and 10 vol. % of DI water, respectively. It is evident that our pressure sensors can work with different types of liquid mediums. It is also interesting to note that the capacitance variations and hence pressure sensitivity depends heavily on the types of liquid medium used as well as the measurement frequency. A complete 3D mapping of the pressure sensitivity versus liquid mediums and frequency will be of great help to optimize the device performances and will be presented in following studies. The device stability was also investigated as shown in Figure 4b. We use pure DI water in this case and tested the sensing stability under 120 Pa and 200 Pa, respectively. The pressure sensor was repeated pressed and released for 500 cycles and the capacitances are very stable showing negligible performance degradations.

## 4. Conclusions

In conclusion, we demonstrated the successful design and fabrication of highly sensitive capillary pressure sensors. We used an innovative 3D printing method to directly and conveniently print out the designed patterns in software. In conventional parallel plate pressure sensors, the capacitance change was due to the pressure-induced interlayer distance variation. Our devices employed distinct pressure sensing mechanisms where the liquid medium in the solution pool will be extruded into the capillary sensing tube between two conductive elastomer electrodes, leading to significant capacitance changes due to the large dielectric constant variations. Our devices showed excellent pressure sensing behaviors when using ethanol, DI water and their mixtures as liquid mediums. A pressure sensitivity as high as 547.9 KPa^−1^ was achieved, representing a significant advance over previous studies. The capillary pressure sensors also showed stable performances with negligible performance degradations after 500 pressing cycles. Our innovative device fabrication methods for pressure sensors with significantly improved device performances are expected to find important applications for future stretchable and wearable electronic systems.

## Figures and Tables

**Table 1:** Sensitivities of PDMS-based pressure sensors.

Device Structures	Sensitivity/KPa^−1^	Response Time/s	Ref.
All-graphene on PDMS	0.002	<0.2	[33]
PDMS waveguide	0.2	0.3	[28]
Micro-lined and micro-pyramid PDMS	0.55	0.2	[20]
Bioinspired porous structure PDMS	0.63	0.01	[21]
Au-coated PDMS micropillars	2	0.05	[11]
PEDOT:PSS coated PDMS micro-pyramids	4.8	0.2	[24]
Graphene coated PDMS microdot array	14	0.03	[23]
ITO/PET coated ionic gels micro-pyramids	41	0.02	[35]
3D printed PDMS capillary sensor	547.9	<1	This work

## Supplementary Materials

The following are available online at http://www.mdpi.com/1424-8220/18/4/1001/s1, Schematic illustration and photographs of device fabrication process, liquid levels upon pressing, other device pattern designs, etc.
