# Supplementary material for: Direct Printing of Stretchable Elastomers for Highly Sensitive Capillary Pressure Sensors
Source: Sensors (Basel). 2018 Mar 28;18(4):1001. doi: 10.3390/s18041001 (PMC5948908; doi:10.3390/s18041001)
Supplement: Supplementary file 1 [file sensors-18-01001-s001.pdf]

Supporting Information

Direct Printing of Stretchable Conductive Elastomers for Capacitive Pressure Sensors

Wenguang Liu[1], and Chaoyi Yan[1,2*]

1. School of Advanced Materials, Shenzhen Graduate School, Peking University, Shenzhen 518005, P. R. China

2. College of Chemistry and Environmental Engineering, Shenzhen University, Shenzhen 518060, P. R. China

**E-mail: yancy@pkusz.edu.cn*

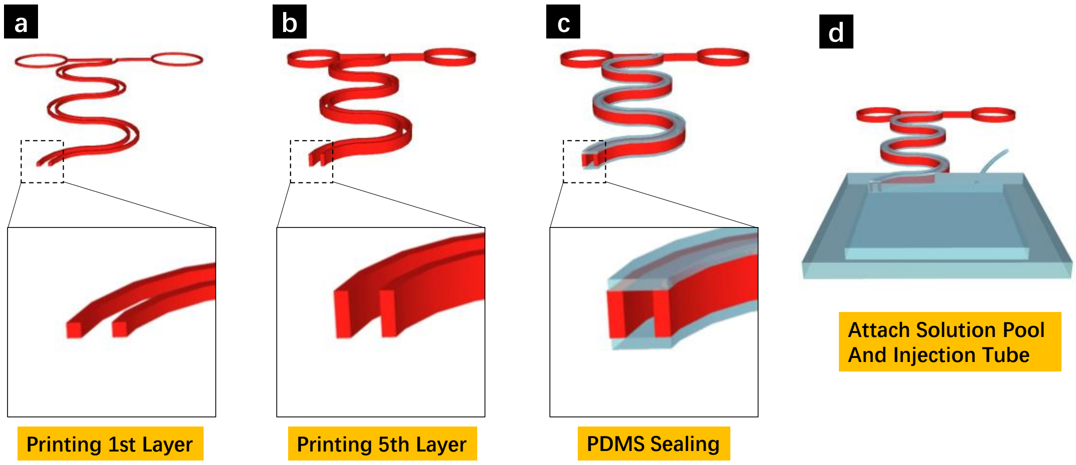

Figure S1. Schematic illustration of the manufacturing process of our capillary pressure sensor. (a) Printing 1st and (b) 5th layer directly on temper glass substrate; (c) Seal the top and bottom of the adjacent conductive electrodes to form the capillary conduction channel; (d) Attach solution pool and injection tube to complete the pressure sensor structure.

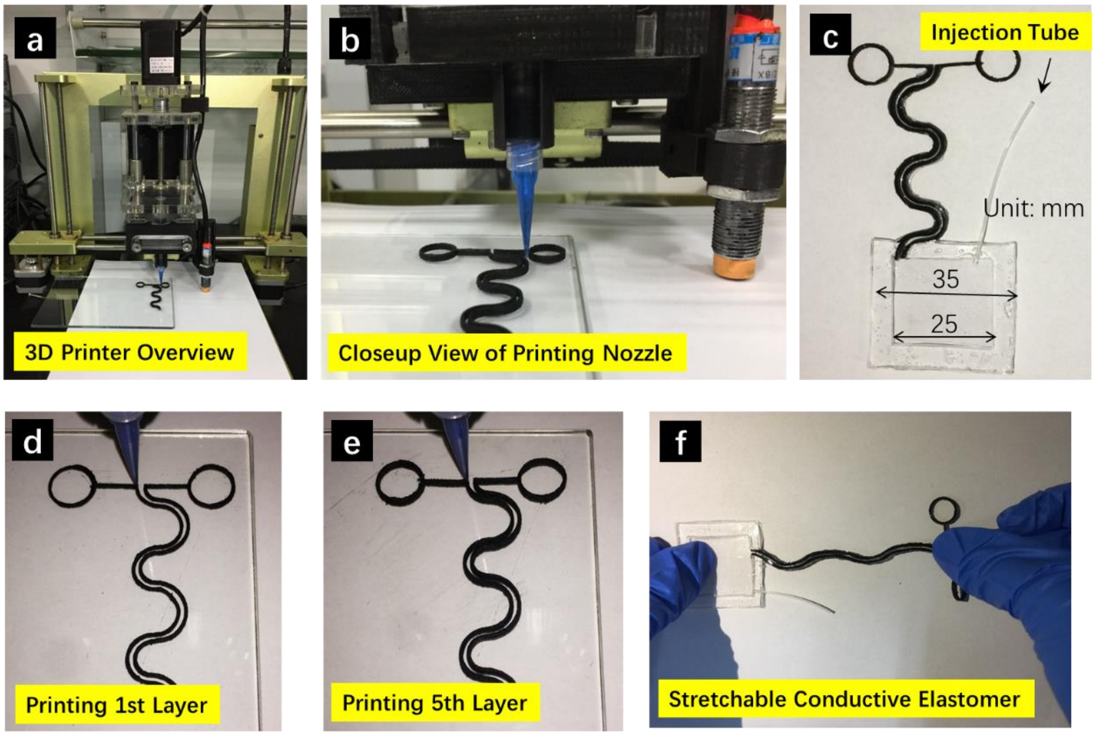

Figure S2. (a) Overview of the home-build 3D printer; (b) Close-up view of printing nozzle for extrusion of conductive CNT/PDMS ink; (c) Photograph of a complete pressure sensor with solution pool and injection tube; (d–e) Photographs of printing 1st layer and 5th layer of the elastomer electrode directly on temper glass substrate; (f) Photograph of the capillary electrodes made of conductive elastomer upon stretching.

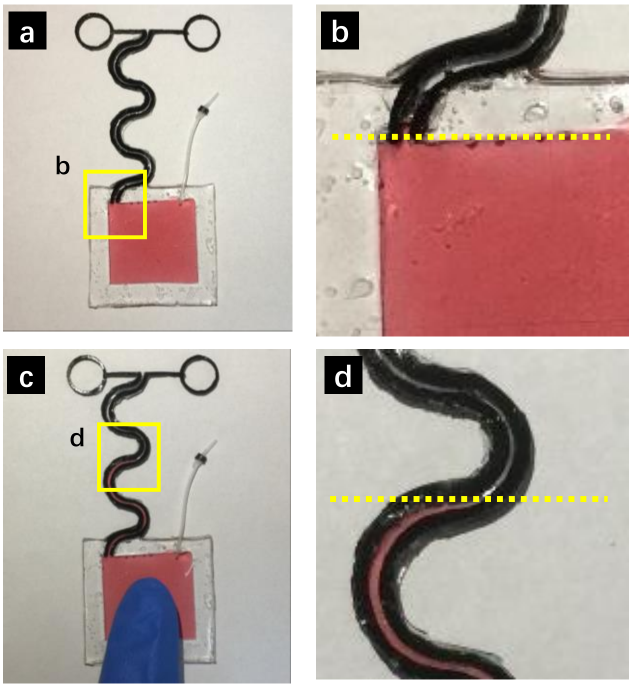

Figure S3. Photographs of the pressure sensor (a-b) without and (c-d) with external pressure. The liquid medium levels can be clearly viewed in the enlarged views.

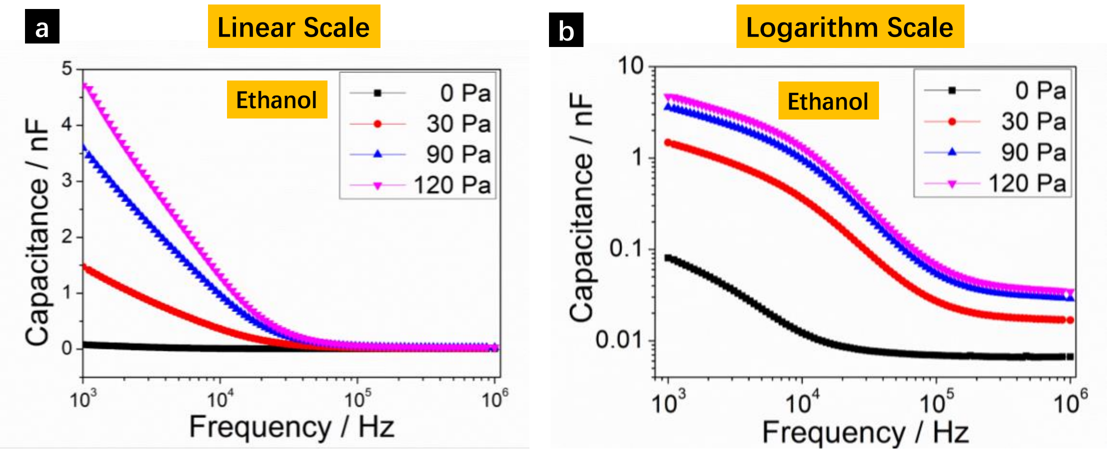

Figure S4. The relationship between capacitance and frequency at applied pressure of 0 Pa, 30 Pa, 90 Pa and 120 Pa within 1 KHz – 1 MHz frequency range: (a) linear scale and (b) logarithm scale plot.

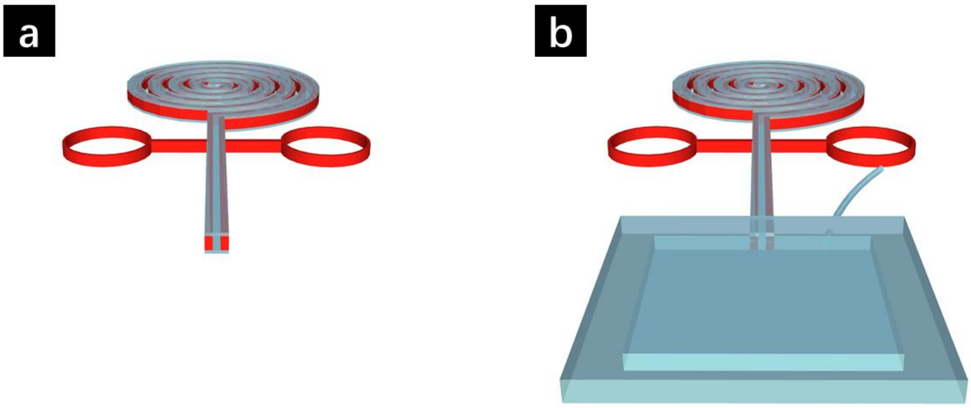

Figure S5. Bi-spiral capillary patterns as more compact designs than the serpentine capillary tubes. The overall length of the capillary tube can be extended within a limited space thus enable the detection over a larger pressure range.

----THE END----
